# Giant negative Poisson's ratio in two-dimensional V-shaped materials†

**DOI:** 10.1039/d1na00212k

**Published:** 2021-06-22

**Authors:** Xikui Ma, Jian Liu, Yingcai Fan, Weifeng Li, Jifan Hu, Mingwen Zhao

**Affiliations:** School of Physics, Shandong University Jinan 250100 Shandong China hujf@sdu.edu.cn zmw@sdu.edu.cn

## Abstract

Two-dimensional (2D) auxetic materials with exceptional negative Poisson's ratios (NPR) are drawing increasing interest due to their potential use in medicine, fasteners, tougher composites and many other applications. Improving the auxetic performance of 2D materials is currently crucial. Here, using first-principles calculations, we demonstrated giant in-plane NPRs in MX monolayers (M = Al, Ga, In, Zn, Cd; X = P, As, Sb, S, Se, Te) with a unique V-shaped configuration. Our calculations showed that GaP, GaAs, GaSb, ZnS and ZnTe monolayers exhibit exceptional all-angle in-plane NPRs. Remarkably, the AlP monolayer possesses a giant NPR of −1.779, by far the largest NPR in 2D materials. The NPRs of these MX monolayers are correlated to the highly anisotropic features of the V-shaped geometry. The exotic mechanical properties of the V-shaped MX monolayers provide a new family of 2D auxetic materials, as well as a useful guidance for tuning the NPR of 2D materials.

## Abstract 2

Two-dimensional (2D) auxetic materials with exceptional negative Poisson's ratios (NPR) are drawing increasing interest due to their potential use in medicine, fasteners, tougher composites and many other applications.

## Graphical abstract

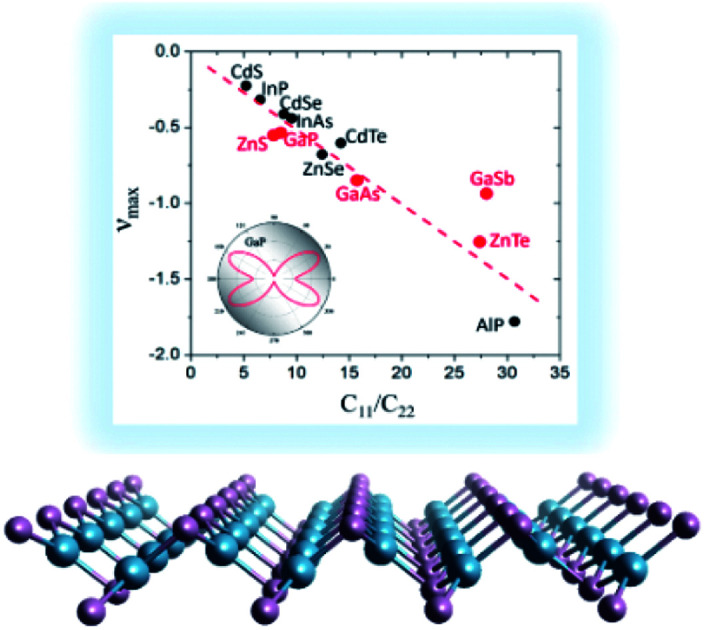

## Introduction

Poisson's ratio which is defined as the ratio of lateral contraction strain to a longitudinal extension strain in the elastic loading direction serves as a fundamental parameter to quantify the mechanical properties of materials.^1^ Most of the materials have positive Poisson's ratios (PPRs), *i.e.*, they would contract (or expand) laterally when stretched (or compressed) longitudinally. As an exceptional scenario, some materials will expand (or contract) laterally under a longitudinal tensile (compression) strain,^2–4^ which leads to a negative Poisson's ratio (NPR). Such auxetic properties have been found in three-dimensional (3D) materials with re-entrant or chiral structures, which hold great promise in a wide range of fields, such as tissue engineering,^5^ medicine,^6^ tougher composites,^7^ fasteners,^8,9^ bulletproof vests, aircraft,^10^ national security,^11^ and so on.^2,12–16^

Two-dimensional (2D) materials have natural advantages in building nanoscale devices. The abundant configurations and exotic electronic states of 2D materials also bring about new concepts for regulating the mechanical properties. The auxetic properties of two-dimensional (2D) material have been predicted according to the Gibson theory.^17^ After the first realization of 2D auxetic effects in graphene with thermally induced ripples,^18^ a number of 2D materials have been reported to have a NPR.^19–30^ For example, monolayer black phosphorene with a buckled structure was predicted to have a NPR of about −0.027 which has been confirmed in experiments.^11,19^ Generally, the auxetic behavior of 2D materials is correlated to the geometric structures and the deformation mechanisms of the materials dominated by the inter-atomic interaction. Some unique geometric configurations, such as re-entrant and hinged structures, have inherent auxetic features independent of atomic interactions.^20–28^ However, in other cases, the auxetic behavior is related to the electronic structures of 2D materials which determine the atomic interactions, rather than the geometry.^29–32^ A strong auxetic effect will greatly improve the mechanical performance of materials, such as dentation resistance or hardness. However, the auxetic effects revealed in 2D materials remain very weak due to the low NPR values. Additionally, the auxetic properties of these 2D auxetic materials are restricted in some specific angle regions. An all-angle in-plane NPR is highly desired.

Here, based on first-principles calculations, we report a new family of 2D auxetic materials, MX (M = Al, Ga, In, Zn, Cd; X = P, As, Sb, S, Se, Te) monolayers, with exceptional auxetic properties. Our calculations showed that the AlP monolayer has the strongest auxetic performance with a NPR of up to −1.779, by far the largest NPR in 2D materials. Additionally, GaP, GaAs, GaSb, ZnS, and ZnTe monolayers exhibit all-angle in-plane auxetic behavior. The excellent auxetic properties of these MX monolayers were ascribed to the synergistic effect of the unique V-shaped configuration and the special deformation mechanism. The correlation between the NPR and elastic constants (*C*_11_ and *C*_22_) was also established. The giant all-angle in-plane NPR in V-shaped MX monolayers opens an avenue for the design of 2D auxetic materials and provides potential for application in nanoscale electromechanical devices.

## Method

The first-principles calculations were carried out by using the density-functional theory (DFT) implemented in the Vienna *ab initio* simulation package (VASP).^33–35^ The electron–ion interaction was described using the projector augmented wave (PAW) approach.^36^ A generalized gradient approximation (GGA)^37^ in the form of Perdew–Burke–Ernzerhof (PBE) for the electron exchange–correlation functional was employed for the structural optimization and electronic structure calculations. The energy cutoff of the plane-wave basis set^38^ was 500 eV. The unit cell was repeated periodically along the *x*- and *y*-directions, while a large vacuum space of 20 Å was applied along the *z*-direction to avoid interaction between adjacent images. The Brillouin zone (BZ) was sampled by using the Gamma method with 11 × 9 × 1 *k*-point sampling for structural optimization. All the atoms were fully relaxed without any symmetry restriction until the residual forces on each atom are smaller than 0.005 eV Å^−1^. The criterion for energy convergence is 10^−7^ eV per cell.

For a 2D material with an orthorhombic lattice, there are four elastic constants: *C*_11_, *C*_12_, *C*_22_, and *C*_66_ which can be evaluated from first-principles calculations.^39^ The orientation-dependent in-plane Young's modulus *Y*(*θ*) and Poisson's ratio *ν*(*θ*) of anisotropic 2D materials are expressed as follows:^40^1
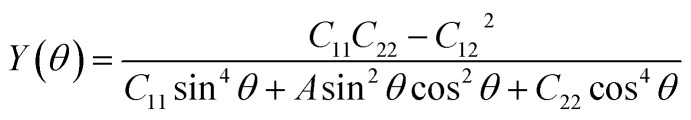
2
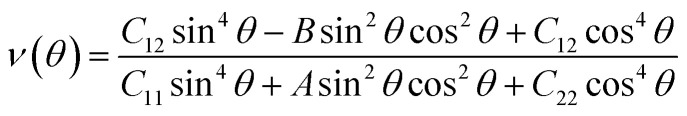
where *A* = (*C*_11_*C*_22_ − *C*_12_^2^)/*C*_66_ − 2*C*_12_, *B* = *C*_11_ + *C*_22_ − (*C*_11_*C*_22_ − *C*_12_^2^)/*C*_66_ and *θ* represents the angle of the applied strain to the *x*-direction. *Y*_*x*_/*ν*_*xy*_ and *Y*_*y*_/*ν*_*yx*_ correspond to *θ* = 0 and 90°, respectively.

## Results and discussion

**Fig. 1:**
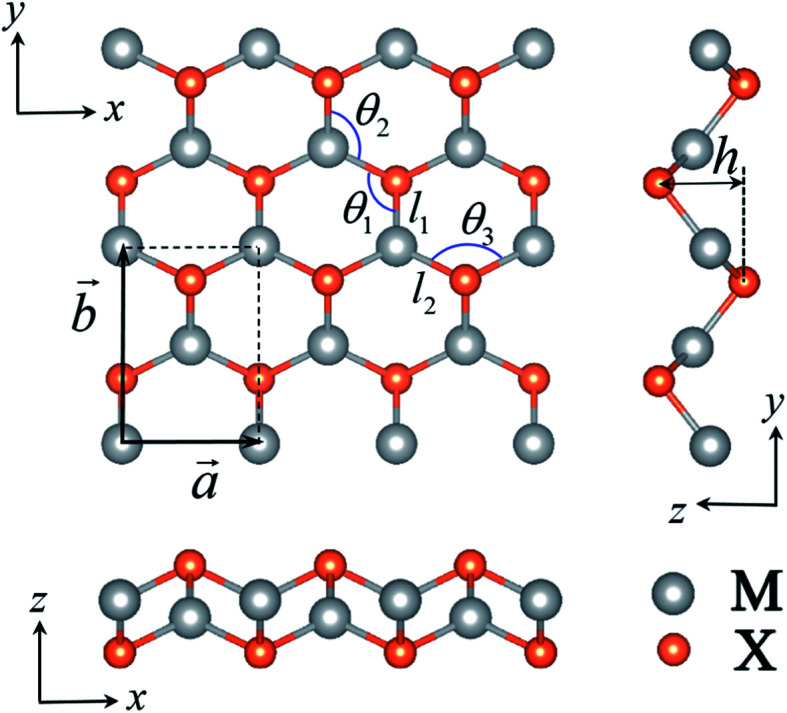
The top and side views of an MX monolayer. The rectangular area represents the primitive cell. The gray and orange balls represent the M and X atoms, respectively.

The atomic structures of the V-shaped MX monolayers considered in this work are plotted in Fig. 1. They contain four atomic layers (X–M–M–X) and can be regarded as a distorted honeycomb lattice with the space group *Pmn*2_1_. Each M atom bonds to three X atoms and *vice versa*. The primitive cell has two M atoms and two X atoms with two orthorhombic basis vectors (*a⃑* and *b⃑*) which are taken as *x*- and *y*-directions, respectively. From the side views of the monolayers, we can see the different atomic arrangements along the *x*- and *y*-directions as shown in Fig. 1, which are expected to bring about different mechanical properties. The optimized structural parameters of the MX monolayers (M = Al, Ga, In, Zn, Cd; X = P, As, Sb, S, Se, Te) are presented in Table 1. The bond angles deviate from the 120° of the standard honeycomb lattice. The thickness (*h*) varies from 1.840 Å to 3.323 Å. Although these V-shaped MX monolayers have not yet been synthesized in experiments, the dynamic stability of these V-shaped monolayers has been verified from the phonon spectra which are free from imaginary frequency modes.^41^ We also checked the stability of the AlP monolayer from the phonon spectrum and molecular dynamics simulations, as shown in Fig. S1 and S2 of the ESI.†

**Table 1:** The lattice constants *a* and *b*, thickness *h*, bond angles *θ*_1_, *θ*_2_, *θ*_3_ and bond lengths *l*_1_ and *l*_2_ of MX monolayers. The lengths are in angstroms; angles are in degrees

	*a*	*b*	*h*	*θ*_1_	*θ*_2_	*θ*_3_	*l*_1_	*l*_2_
AlP	3.808	5.445	2.394	124.648	93.130	110.125	2.300	2.324
GaP	3.827	5.897	2.159	124.762	98.990	109.934	2.319	2.337
InP	4.412	6.347	2.443	125.400	97.741	108.847	2.527	2.545
GaAs	3.959	5.712	2.624	126.088	92.290	107.621	2.434	2.452
InAs	4.254	6.183	2.853	126.495	92.341	106.918	2.631	2.648
GaSb	4.259	5.344	3.323	127.027	82.263	105.893	2.657	2.668
ZnS	3.796	6.000	1.840	123.450	103.897	112.909	2.261	2.277
ZnSe	3.960	5.815	2.419	124.980	95.737	109.994	2.396	2.468
ZnTe	4.420	5.860	2.898	125.504	90.139	108.989	2.594	2.617
CdS	4.128	6.530	2.167	122.902	102.809	116.077	2.236	2.437
CdSe	4.267	6.358	2.623	125.425	96.110	109.119	2.598	2.623
CdTe	4.550	6.179	3.101	125.944	90.678	108.140	2.782	2.809

**Fig. 2:**
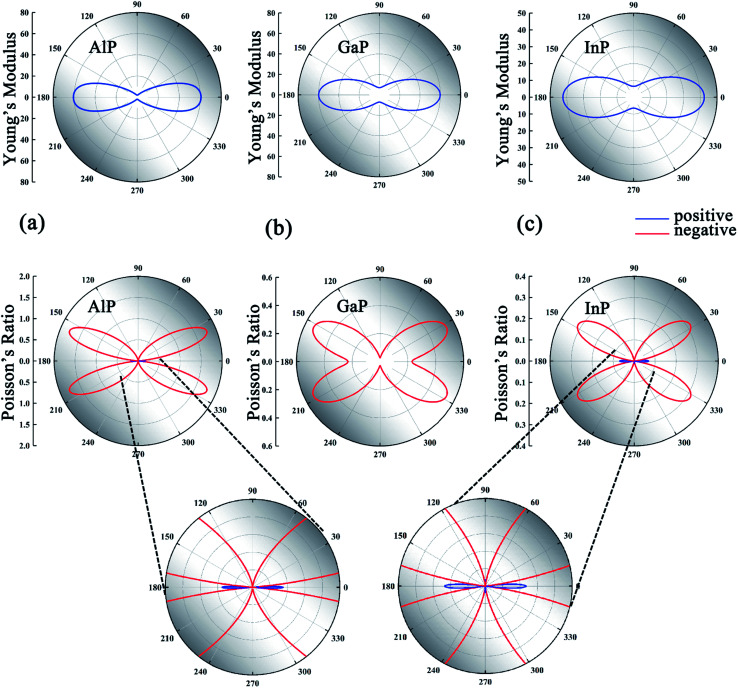
(a)–(c) The mechanical properties of AlP, GaP and InP monolayers. The upper and lower rows represent the Young's modulus and Poisson's ratio of AlP, GaP and InP monolayers, respectively. The lowest row is the enlarged view of the Poisson's ratio for AlP and InP monolayers. The NPR and PPR are marked with red and blue lines, respectively.

The mechanical parameters including the Young's modulus and Poisson's ratio of the MX monolayers calculated from first-principles calculations are presented in Table 2. The elastic constants satisfy the mechanical stability criteria^39^*C*_11_*C*_22_ − *C*_12_^2^ > 0 and *C*_66_ > 0, confirming the stability of these MX monolayers. Notably, these MX monolayers show highly anisotropic mechanical properties. The Young's modulus along the *x*-direction (*Y*_*x*_) is much higher than that along the *y*-direction (*Y*_*y*_) for all the monolayers. The *Y*_*x*_/*Y*_*y*_ even exceeds 30 in the AlP monolayer. Such high anisotropic features can be ascribed to the different deformation mechanisms along the *x*- and *y*-direction in the V-shaped configuration due to the different atomic alignments. The Young's modulus reaches the maximum value in the *x*-direction (*θ* = 0°, 180°), while the minimal value appears in the *y*-direction (*θ* = 90°, 270°), as shown in Fig. 2. The Young's moduli of these MX monolayers (1.23–60.72 N m^−1^) are much smaller than those of graphene (354.00 N m^−1^)^42^ and phosphorene (103.32 N m^−1^),^43^ suggesting that they can be easily deformed by external strains.

**Table 2:** The mechanical parameters of the MX monolayers obtained from first-principles calculations. The elastic constants and Young's moduli are in N m^−1^ and the angles of the maximal NPR (*θ*_c_) are in degrees. The Poisson's ratios were calculated from the elastic constants, while the data in parentheses were obtained from the response to an external strain

	*C*_11_	*C*_22_	*C*_12_	*C*_66_	*Y*_*x*_	*Y*_*y*_	*ν*_max_	*θ*_c_	*ν*_*xy*_	*ν*_*yx*_
AlP	60.778	1.981	0.347	14.540	60.717	1.979	−1.779	23.5	0.175 (0.185)	0.006 (0.005)
GaP	60.203	7.108	−1.632	13.268	59.828	7.063	−0.536	27.5	−0.230 (−0.233)	−0.027 (−0.030)
InP	42.369	6.465	0.444	9.650	42.339	6.460	−0.317	33.2	0.069 (0.067)	0.010 (0.014)
GaAs	55.342	3.511	−0.753	9.651	55.181	3.501	−0.852	25.2	−0.214 (−0.201)	−0.014 (−0.014)
InAs	39.887	4.178	0.723	7.709	39.762	4.165	−0.44	31.5	0.173 (0.176)	0.018 (0.021)
GaSb	48.659	1.733	−1.071	4.198	47.998	1.710	−0.939	18.3	−0.618 (−0.606)	−0.022 (−0.024)
ZnS	42.999	5.425	−0.420	11.096	42.967	5.421	−0.550	29.8	−0.077 (−0.075)	−0.010 (−0.010)
ZnSe	41.558	3.339	0.358	8.279	41.520	3.336	−0.678	28.7	0.107 (0.109)	0.009 (0.009)
ZnTe	39.113	1.430	−0.054	5.762	39.111	1.430	−1.257	23.5	−0.038 (−0.037)	−0.001 (−0.003)
CdS	26.693	5.122	1.054	6.799	26.477	5.080	−0.224	36.7	0.206 (0.210)	0.040 (0.041)
CdSe	26.286	2.986	1.215	5.599	25.791	2.930	−0.412	33.8	0.407 (0.415)	0.046 (0.047)
CdTe	26.321	1.851	0.739	4.358	26.026	1.830	−0.602	29.8	0.399 (0.370)	0.028 (0.028)

The Poisson's ratios along the *x*-direction (*ν*_*xy*_) and *y*-direction (*ν*_*yx*_) derived from the elastic constants *C*_11_, *C*_22_ and *C*_12_ also have large differences in magnitude which lead to highly anisotropic characteristics of the Poisson's ratios. Although the Poisson's ratios along *x*- and *y*-directions (*ν*_*xy*_, *ν*_*yx*_) of these MX monolayers are small and even positive, NPRs emerge in all the MX monolayers at all or most angles, as shown in Fig. 2 and S3 of the ESI.† The maximal NPR (*ν*_max_) of the MX monolayers and the corresponding angle *θ*_c_ are presented in Table 2. Remarkably, the Poisson's ratio of the AlP monolayer reaches an unprecedented value of −1.779 at *θ* = 23.5°, significantly larger than those of the 2D auxetic materials proposed in previous literature, such as α-phosphorene (−0.027),^11^ SiC_6_ (−0.042),^20^ δ-phosphorene (−0.267),^44^ Be_5_C_2_ (−0.16),^21^ W_2_C (−0.4),^23^ 1T-MX_2_ (−0.03 to −0.37),^29^ AgCl (−0.18)^31^ and Ag_2_S (−0.52).^45^ It is interesting to see that InP, GaAs, GaSb, ZnS, and ZnTe monolayers possess all-angle in-plane NPRs. Such giant all-angle NPRs hold great promise for nanoscale mechanical devices.

**Fig. 3:**
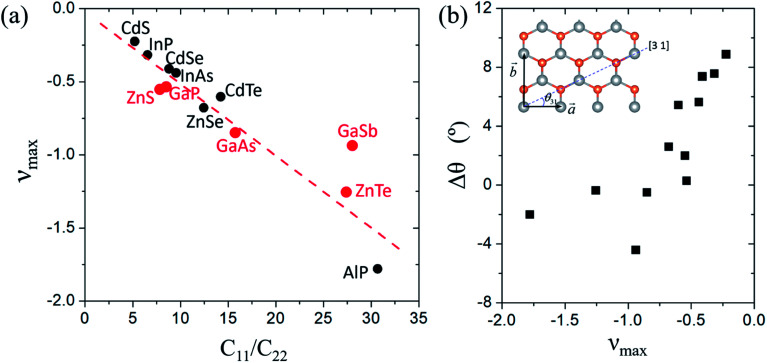
(a) The relationship between the maximal NPR (*ν*_max_) and the elastic constant ratio *C*_11_/*C*_22_ of the MX monolayer. The red dots indicate the MX monolayers with all-angle NPRs. The dashed line indicates the fitting data of *y* = −0.0528*x* + 0.01. (b) The deviation of the direction with the maximal NPR (*θ*_c_) relative to the [3 1] direction (*θ*_31_), Δ*θ* = *θ*_c_ − *θ*_31_, of the MX monolayers.

We correlated the emergence of maximal NPRs to the highly anisotropic elastic constants of the MX monolayers. We adopted the ratio of *C*_11_/*C*_22_ to characterize the anisotropic elastic constants. The relationship between *ν*_max_ and *C*_11_/*C*_22_ of these MX monolayers is plotted in Fig. 3(a). It is found that |*ν*_max_| increases with the increase of *C*_11_/*C*_22_, exhibiting a nearly linear correlation. The AlP monolayer has the largest *C*_11_/*C*_22_ and thus the strongest NPR effect. The relationship between the NPR and *C*_11_/*C*_22_ offers a general strategy for the design of auxetic materials.

Notably, the NPRs reported in previous literature are always accompanied by negative *C*_12_ values. However, some of the auxetic MX monolayers considered in this work have positive *C*_12_ values, as listed in Table 2. The origins of the NPR in these materials can be qualitatively demonstrated from eqn (2). Considering the mechanical stability requirement (*C*_11_*C*_22_ − *C*_12_^2^) > 0 and *C*_66_ > 0 of the MX monolayers, the emergence of a negative Poisson's ratio is dominated by the sign of the numerator of eqn (2), because of the positive definite denominator. For highly anisotropic MX materials, supposing *C*_11_ ≫ *C*_22_, *C*_66_ > *C*_22_, we get a positive *B* parameter in eqn (2), which leads to a negative Poisson's ratio at specific angle regions independent of the sign of *C*_12_. Therefore, a negative *C*_12_ is not a requisite for highly anisotropic 2D auxetic materials.

It is interesting to see the relationship between the strongest NPR direction and the [3 1] direction of the MX monolayers. From Fig. 3(a), we can find that the strongest NPR direction is very close to the [3 1] direction with a deviation of less than 8 degrees. For the MX monolayers with large *ν*_max_, *e.g.* AlP, ZnTe and GaAs, the deviations (Δ*θ*) are even less than 2 degrees. This implies the special deformation mechanism of the MX monolayers along the [3 1] direction, which is quite crucial for the utilization of the NPR effect.

Poisson's ratio (*ν*_*ij*_) along the *i*-direction can also be determined from the response of materials along the *j*-direction (*ε*_*j*_) under uniaxial strain (*ε*_*i*_) along the *i*-direction, *ν*_*ij*_ = −*ε*_*j*_/*ε*_*i*_. We recalculated the Poisson's ratios along *x*- and *y*-directions using this strategy. The uniaxial strain ranging from −3% to 3% is considered in our calculations. The response of AlP, GaP and InP monolayers to the uniaxial strains is shown in Fig. S4 of the ESI.† It is found that these MX monolayers show elastic behavior in the strain range reflected by the linear *ε*_*x*_ − *ε*_*y*_ relationship. The Poisson's ratios determined from this strategy are in good agreement with those from the elastic constant calculations, as listed in Table 2.

**Fig. 4:**
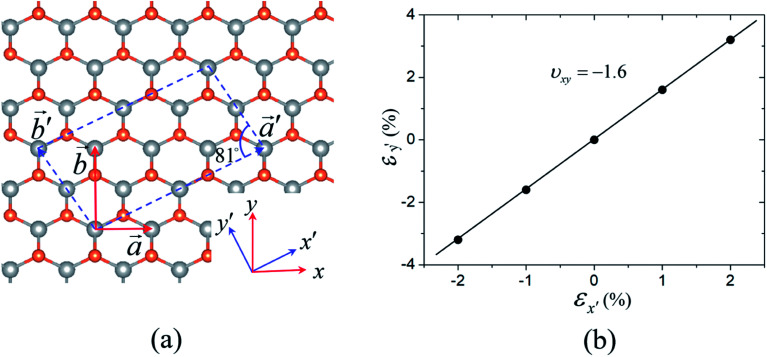
(a) The large supercell (indicated by the dashed lines) used for determining the NPR of the AlP monolayer along the [3 1] direction. (b) The response to strain along the *y*′-direction as a function of applied strain along the *x*′-direction with 1% and 2%. The labels of axes *x*′ and *y*′ are shown in Fig. 5(a).

We also evaluated the Poisson's ratio of the AlP monolayer along the [3 1] direction with *θ* = 25.5° which is very close to the maximal NPR direction (*θ*_c_ = 23.5°) using the above strategy. We constructed a large supercell with the basis vectors of 
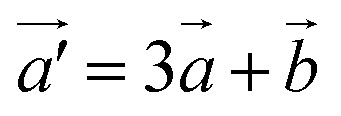
 and 
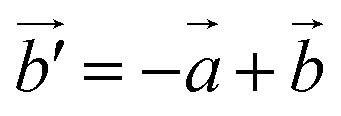
, as shown in Fig. 4(a). Notably, unlike the basis vectors of the primitive cell, the two basis vectors of the supercell are not orthogonal. We applied a tensile strain (*ε*_*x*′_) to the supercell along the 
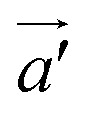
 direction (taken as the *x*′-direction) and then optimized the length and direction of 
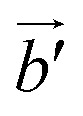
 to determine the response of the supercell (*ε*_*y*′_) along the direction perpendicular to 
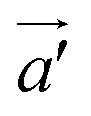
 (taken as the *y*′-direction), as shown in Fig. 4(b). The NPR evaluated from this strategy is about −1.6, in good agreement with that (∼−1.7) calculated from the elastic constants in this direction, confirming the giant NPR of the AlP monolayer. We also adopted the HSE functional to check the NPR of the AlP monolayer, as shown in Fig. S5 of the ESI.† It is found that the NPR (∼1.7) obtained from the HSE functional is very close to that from the PBE functional.

**Fig. 5:**
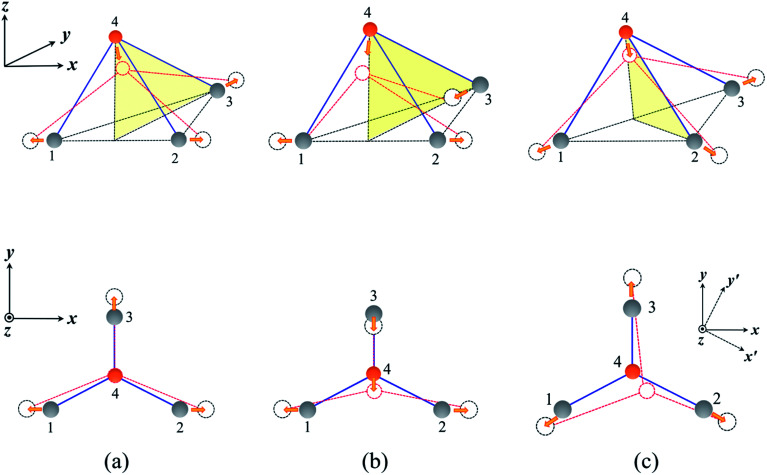
The different deformation mechanisms for NPRs (a) and PPRs (b) in V-shaped MX monolayers. (c) The deformation mechanism when an external strain is applied along the [3 1] direction.

The highly anisotropic mechanical properties of the MX monolayers are closely related to the V-shaped configuration which leads to different deformation mechanisms in response to external strain, as shown in Fig. 5. For simplification, we adopted a tetrahedral mode formed by three M atoms and one X atom to demonstrate the relevant deformation mechanisms. As a tensile strain is applied along the *x*-direction, the bonds (1–4 and 2–4) between M and X atoms are stretched, pulling the X(4) atom downward, as shown in Fig. 5(a) and (b). The response of the bond between atom 3 and 4 determines the sign of the Poisson's ratio. If the bond is compressed, a NPR will be obtained, as shown in Fig. 5(a). Otherwise, we will get a positive Poisson's ratio, as shown in Fig. 5(b). As a strain is applied along the *y*-direction which preserves the mirror symmetry of the lattice, the bond between 3 and 4 is stretched, and the movement of the X atom compresses or stretches the 1–4 and 2–4 bonds. However, the response is weaker than that to the external strain along the *x*-direction, which leads to different elastic constants (*C*_11_ and *C*_22_). Notably, the three M–X bonds are not equivalent in the V-shaped configuration of the MX monolayers. For the AlP monolayer, the bond between 3 and 4 is shorter than the other two bonds by about 0.024 Å. Therefore, when an external strain is applied along the *x*′-direction, the mirror symmetry is broken, and the corresponding deformation differs significantly from the response to the strain along the *y*-direction, as shown in Fig. 5(c).

The variation of the NPR of these MX monolayers indicates that the binding characteristics also contribute to the deformation mechanism. From Table 2, we can see that for the MX monolayers, the |*ν*_max_| decreases as M changes from Al to In in the same group of the periodic table. For the ZnX and CdX (X = S–Te) monolayers, however, the |*ν*_max_| decreases as the chalcogen varies from S to Te, suggesting the different mechanisms between these two groups. We have also plotted the orbital-resolved electron density of states and band structures of these MX monolayers in Fig. S6 and S7 of the ESI.† It is found that the p-orbitals of the M and X atoms hybridize in the region near the Fermi level, implying the covalent bonding features of the M–X bonds. Nevertheless, we cannot correlate the auxetic properties directly to the electronic structures of the MX monolayers. The unique NPR behavior of the MX monolayer arises from the synergistic effect of the V-shaped geometry and the M–X binding characteristics.

## Conclusion

We demonstrate from the first-principles calculations the giant in-plane NPRs of the MX (M = Al, Zn, Ga, Cd, In; X = P, As, Sb, S, Se, Te) monolayers. We ascribe the auxetic behavior of the MX monolayers to the unique V-shaped configuration and the special deformation mechanism which lead to strongly anisotropic elastic constants. GaP, GaAs, GaSb, ZnS and ZnTe monolayers possess all-angle in-plane NPRs. Remarkably, an unprecedented value of NPR of −1.779 is found in the AlP monolayer at a specific angle (*θ* = 23.5°), which indicates giant auxetic behavior under uniaxial strain. The maximal NPR |*ν*_max_| can be correlated linearly to the ratio of *C*_11_/*C*_22_ which characterizes the anisotropic features of the materials. The exotic mechanical properties of the V-shaped MX monolayer provide a new family of 2D auxetic materials, as well as a useful guidance for tuning the NPR of 2D materials.

## Conflicts of interest

The authors declare no competing financial interest.

## Supplementary Material

NA-003-D1NA00212K-s001
