# Supplementary material for: Giant negative Poisson's ratio in two-dimensional V-shaped materials
Source: Nanoscale Adv. 2021 Jun 22;3(15):4554–60. doi: 10.1039/d1na00212k (PMC9417312; doi:10.1039/d1na00212k)
Supplement: NA-003-D1NA00212K-s001 [file NA-003-D1NA00212K-s001.pdf]

Electronic Supplementary Material (ESI) for Nanoscale Advances.
This journal is © The Royal Society of Chemistry 2021

Supporting information for

Giant negative Poisson's ratio in two-dimensional V-shaped materials

Xikui Ma, Jian Liu, Yingcai Fan, Weifeng Li, Jifan Hu*, Mingwen Zhao*

School of Physics, Shandong University, Jinan 250100, Shandong, China

Corresponding authors, hujf@sdu.edu.cn (J. Hu), zmw@sdu.edu.cn (M.Zhao)

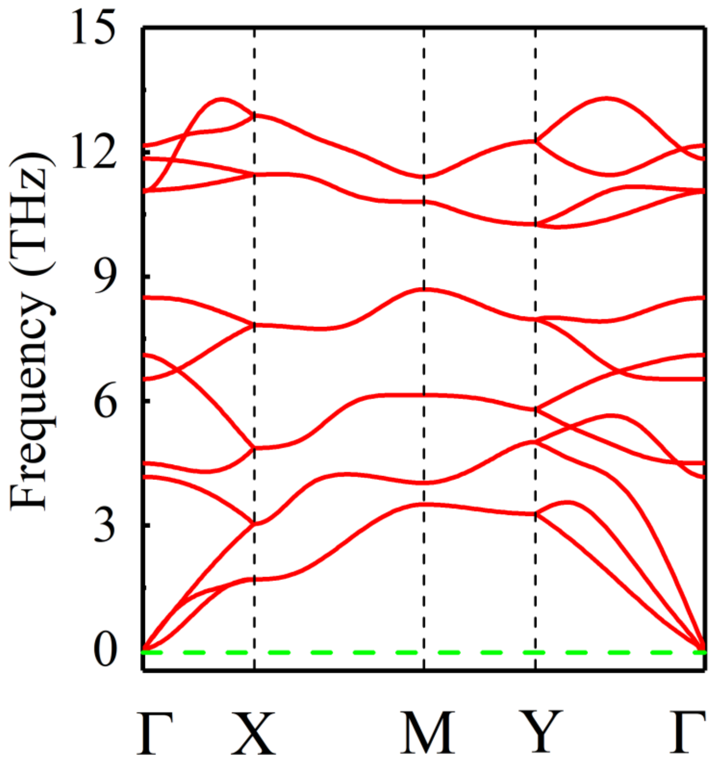

Fig. S1 The phonon spectrum of AlP monolayer.

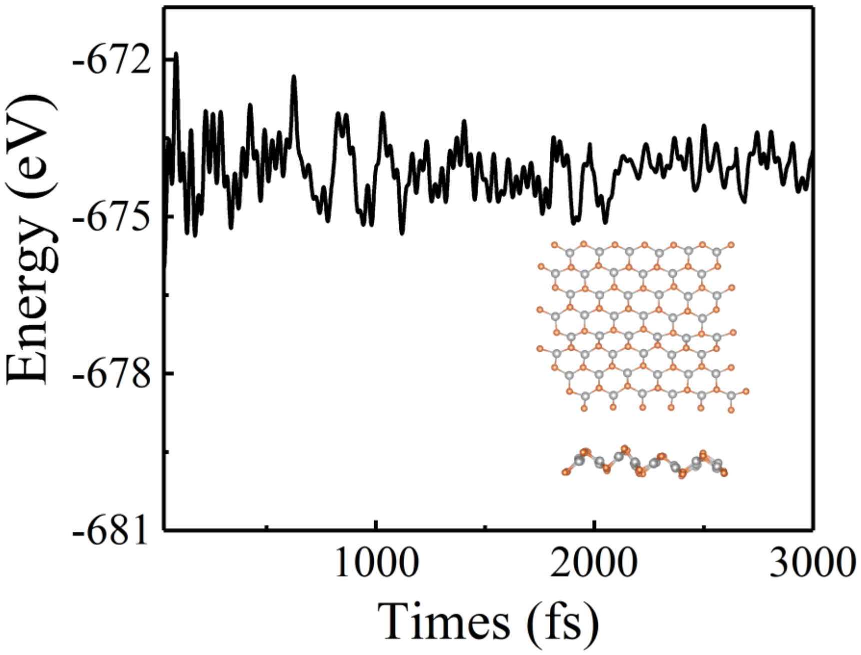

Fig. S2 The total energy fluctuations with respect to the simulation time at 300 K. the geometry of AlP monolayer after 3000 fs is shown in the inset.

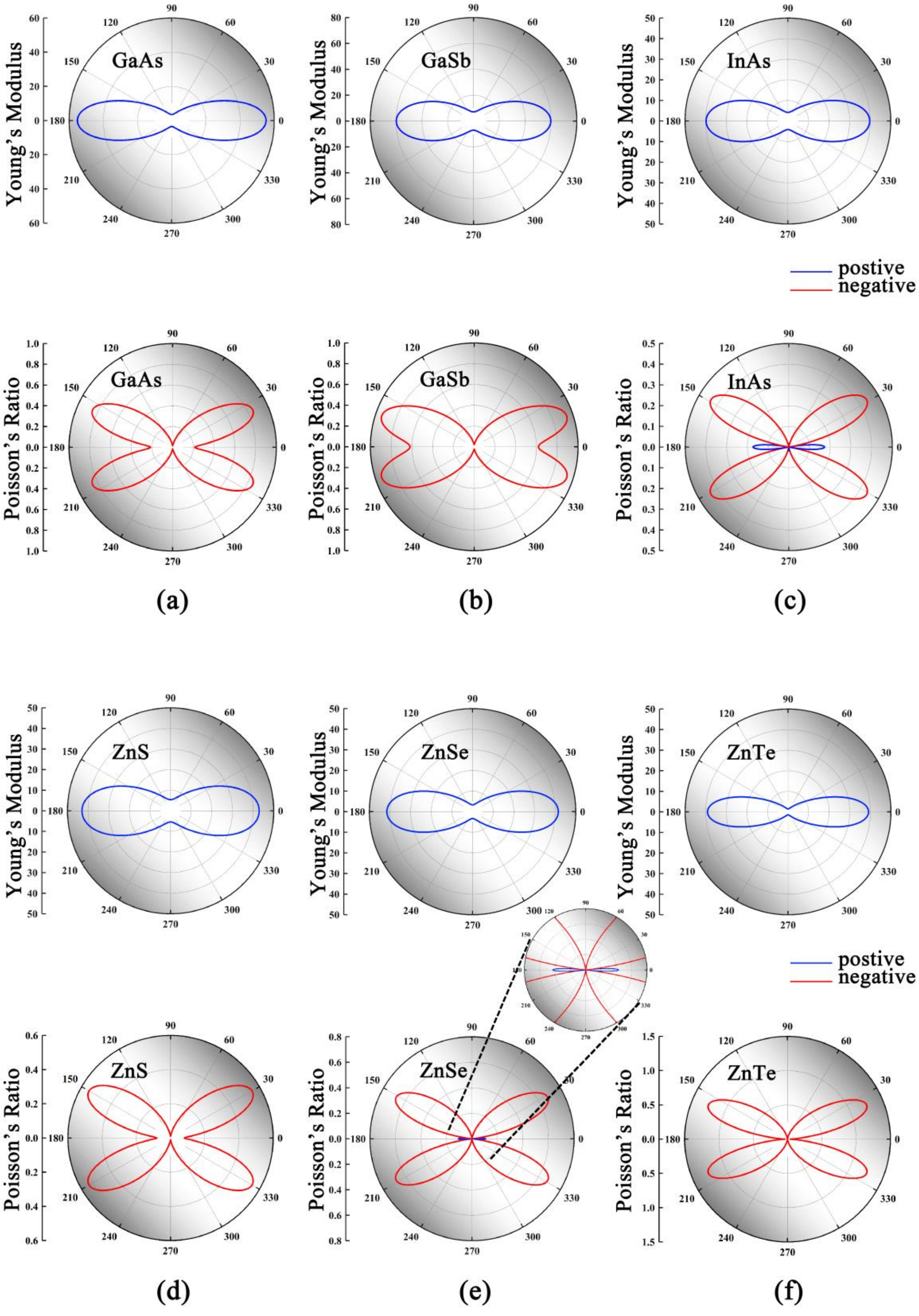
GaAs
GaSb
InAs
ZnS
ZnSe
ZnTe
Young's Modulus
Poisson's Ratio
postive
negative
(a)
(b)
(c)
(d)
(e)
(f)

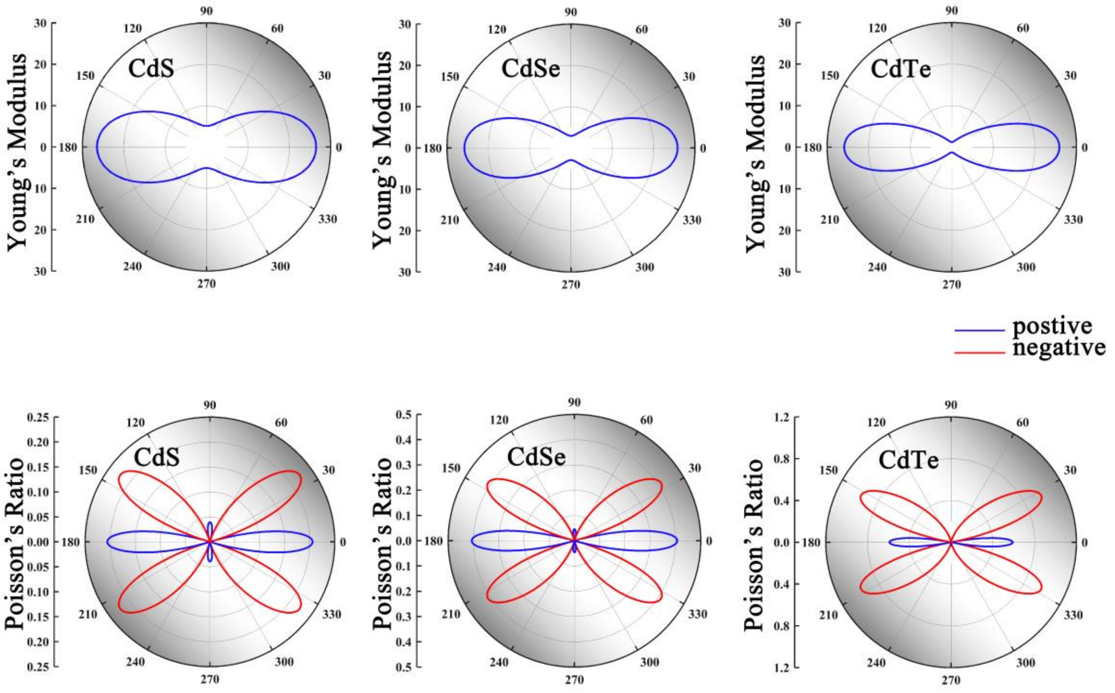

(g) (h) (i)

Fig. S3 (a)-(i) The mechanical properties of GaAs, GaSb, InAs, ZnS, ZnSe, ZnTe, CdS, CdSe and CdTe monolayers. The upper and lower row represent the Young's modulus and Poisson's ratio, respectively, and the middle row is the larger view of Poisson's ratio of ZnTe monolayer. The NPR and PPR are marked with red and blue line, respectively.

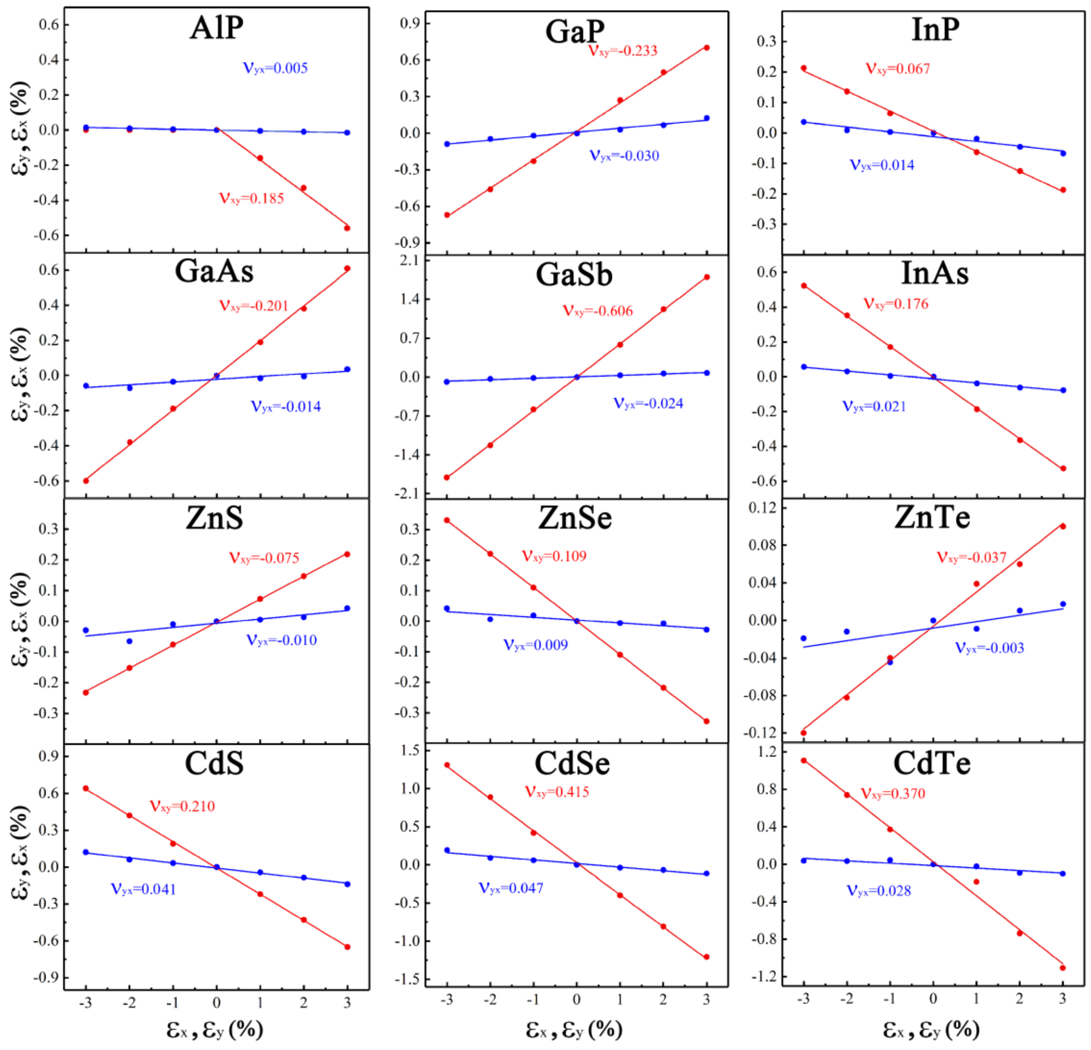

Fig. S4. The response of strain along *y*- (and *x*-) direction when applying a strain from -3% to 3% along *x*- (and *y*-) direction of the MX monolayers.

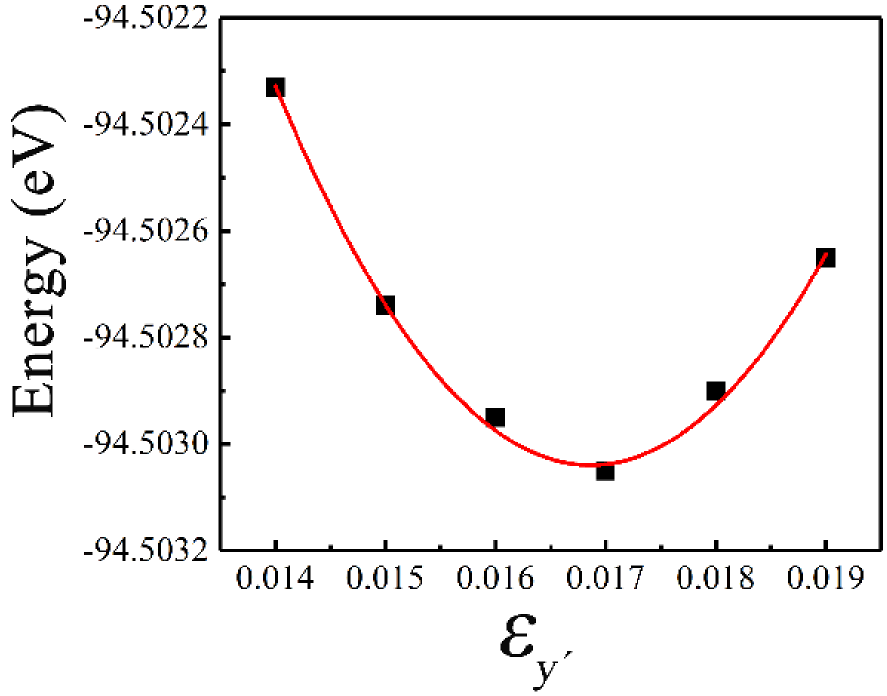

Fig. S5 The relative of energy of structure and difference strain along y' direction when a tensile of 1% was applied along x' direction.

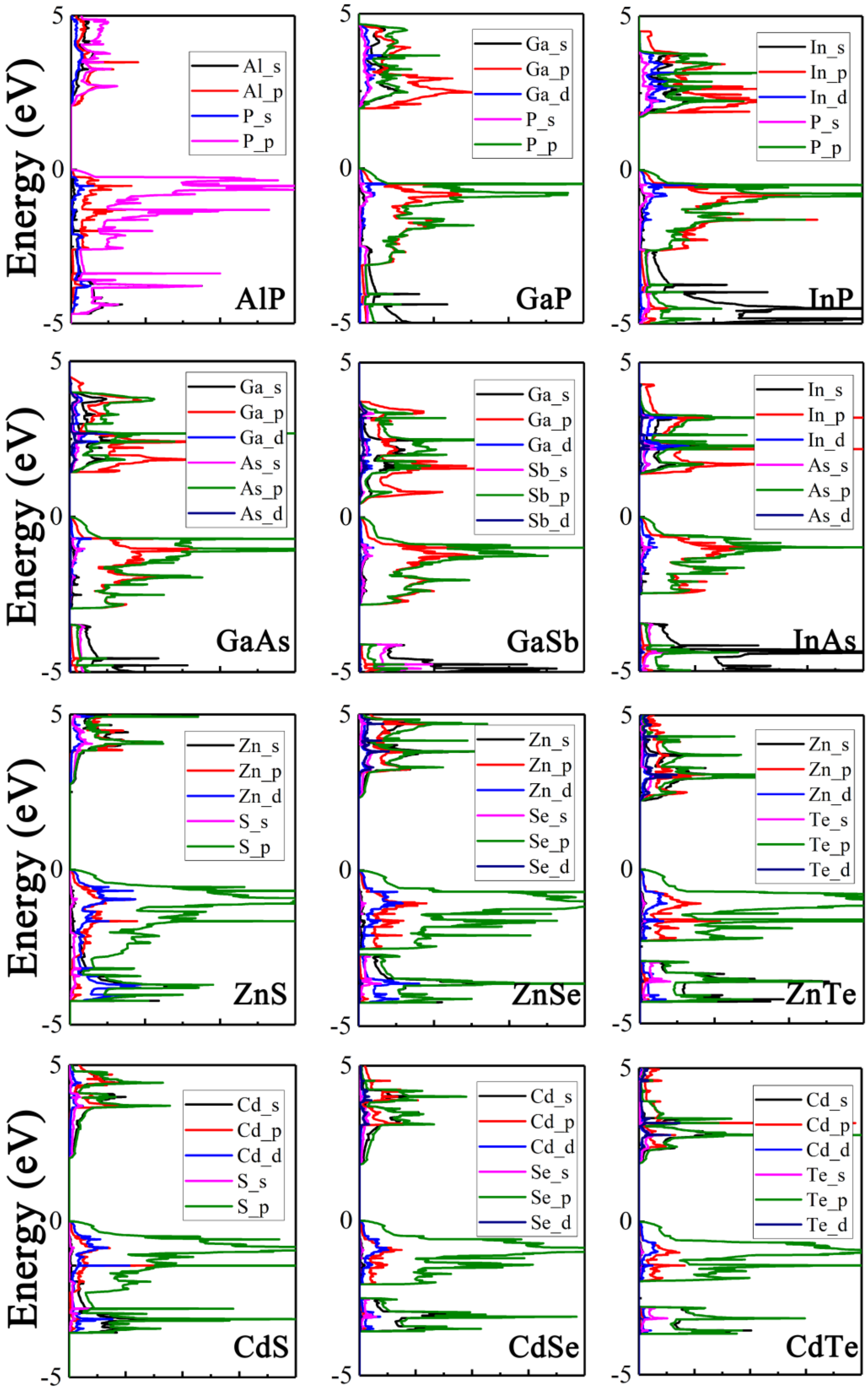

Fig. S6 The orbital-resolved electron density of states (DOS) of the V-shaped MX monolayers. The energy at the Fermi level is set to zero

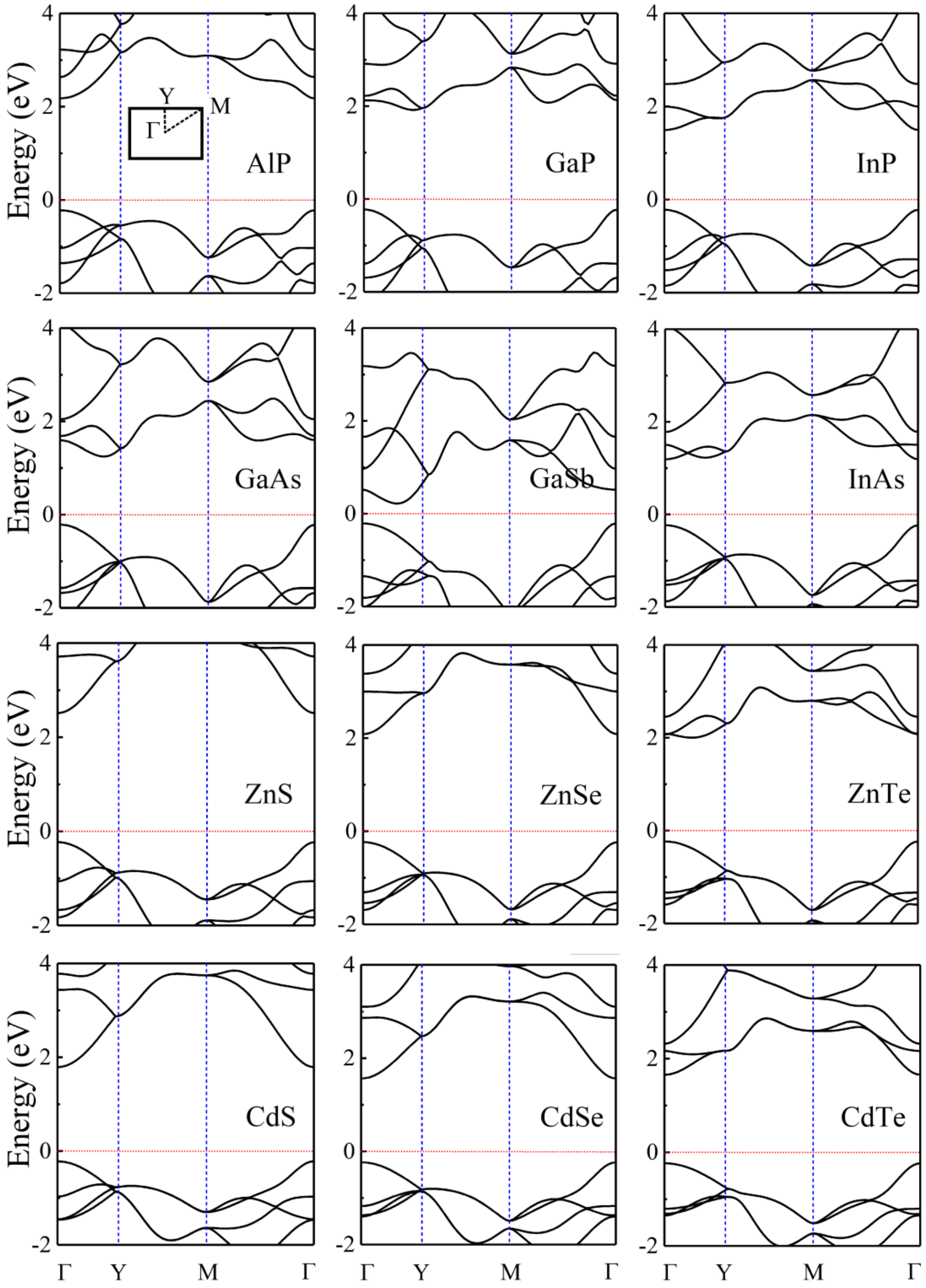

Fig. S7 The band structure of the V-shaped MX monolayers. The energy at the Fermi level is set to zero
